# The Power of a Belief System: A Systematic Qualitative Synthesis of Spiritual Care for Patients with Brain Tumors

**DOI:** 10.3390/jcm13164871

**Published:** 2024-08-18

**Authors:** Neel H. Mehta, Megh Prajapati, Rishi Aeleti, Kush Kinariwala, Karina Ohri, Sean McCabe, Zachary Buller, Sandra Leskinen, Noah L. Nawabi, Vatsal Bhatt, Keval Yerigeri, Vivek Babaria

**Affiliations:** 1 Department of Neurosurgery, Massachusetts General Hospital and Harvard Medical School, Boston, MA 02115, USA; 2 Department of Biomedical Engineering, Cornell University, Ithaca, NY 14850, USA; mp852@cornell.edu; 3 Department of Biology, Cornell University, Ithaca, NY 14850, USA; rr47@cornell.edu; 4 Texas College of Osteopathic Medicine, Fort Worth, TX 7107, USA; kushkinariwala@gmail.com; 5 Department of Pediatrics, Norton College of Medicine, SUNY Upstate, Syracuse, NY 13210, USA; ko335@cornell.edu; 6 Division of Hematology and Oncology, Department of Medicine, Massachusetts General Hospital Cancer Center, Boston, MA 02114, USA; smccabe@mgh.harvard.edu; 7 Department of Chemistry, Harvard University, Cambridge, MA 02138, USA; zachary.buller@college.harvard.edu; 8 State University of New York Downstate Medical Center, New York, NY 11203, USA; sandra.leskinen@downstate.edu; 9 College of Medicine, Medical University of South Carolina, Charleston, SC 29425, USA; nnawabi@bwh.harvard.edu; 10 Department of Medicine, Brigham and Women’s Hospital, Boston, MA 02115, USA; vbhatt1@bwh.harvard.edu; 11 Department of Internal Medicine-Pediatrics, Case Western Reserve University/The MetroHealth System, Cleveland, OH 44109, USA; 12 Orange County Spine and Sports, Interventional Physiatry, Newport Beach, CA 92660, USA; drvivekbabaria@gmail.com

**Keywords:** spirituality, perspective, brain tumors, medicine

## Abstract

**Background**: Diagnosis with a brain tumor is a critical event in the lives of patients and their families due to poor medical prognoses and complex clinical care. Spiritual care interventions have been known to have meaningful effects in morbid diagnoses and palliative medicine, but their role in the neuro-oncologic patient’s experience is poorly understood. This systematic review explores the role of spirituality and its relevance to patient care in the diverse setting of brain tumors. **Methods**: A comprehensive systematic review was conducted following PRISMA-SR guidelines. PUBMED was queried for studies on spirituality and neuro-oncology. Identified studies included RCTs, interviews, surveys, and case reports that examined spirituality in neuro-oncological clinical care, quality of life, and patient experience. Of 214 articles identified, 21 studies met the inclusion criteria, and the results were narratively synthesized. **Results**: Spirituality may play a significant role in mental well-being by reconciling existential questions faced by both patients and caregivers, and can serve as a valuable resource to improve mental well-being and reduce rates of palliative caregiver burnout. However, the paucity of studies examining the education and integration of spiritual awareness within the clinical literature warrants further study. **Conclusions**: While spiritual care interventions may improve the quality of life and mental wellness of patients and their caregivers, it is unclear how spiritual awareness and education should best be implemented. Further research is needed to better understand how key components of spiritual awareness can be integrated into medical education to deepen the patient–physician relationship and improve clinical experiences.

## 1. Introduction

Approximately 90,000 patients in the United States are diagnosed with primary brain tumors each year; when metastatic, this diagnosis is one of the most fatal cancers in the nation [1]. According to the Central Brain Tumor Registry of the United States, malignant brain and central nervous system (CNS) tumors have a five-year relative survival rate of just under 36% [1]. Indeed, malignant peripheral nervous tumors and malignant brain tumors have been noted to result in the greatest years of potential life lost at 20.65 and 19.93 years, respectively [2]. With commonly presenting symptoms ranging from mild headaches and fatigue to severe cognitive impairment or seizures [3], CNS tumors can range from completely benign to highly malignant, and clinical discussions often include intense conversations about lasting cognitive deficits, recurrence or progression, and in severe cases, end-of-life care [4,5]. Dealing with such a life-threatening diagnosis can pose significant challenges for both clinicians treating symptoms and patients facing concerns of mortality. The medical and surgical complexity of these cases often contributes to an even greater level of uncertainty surrounding a patient’s individual response. Ultimately, these factors often compound to exacerbate the mental and emotional burden of such a diagnosis. In this setting, spiritual care has been considered an avenue to achieve truly holistic treatment for patients and their families that alleviates some of those burdens.

Spirituality is a multifaceted concept that is generally defined as the search for meaning, purpose, and connection to something greater than oneself [6]. Unlike religious practices, spirituality is often considered a deeply personal, subjective experience that may or may not involve organized religion. In the medical and surgical spheres, spirituality and spiritual well-being have long been considered essential sources of support for many patients, showing significant positive correlations with quality-of-life measures [7]. Specifically with patients dealing with brain tumor diagnoses, the completion of programs centered on existential meaning-making has been associated with lower rates of depression and higher levels of well-being [8]. This is particularly important for patients with brain tumors and their caregivers who may experience a heightened sense of vulnerability, likely due to the emphasized sense of self-threat, social stigmas, and risk of permanent debilitation that accompany neuro-oncological diagnoses [9,10,11]. Although recognizing spirituality as a key component of care can encourage healthcare teams to prioritize supportive care in conjunction with the surgical management of CNS tumors, there has been little systematic evaluation of spirituality in neuro-oncological patients and their families.

To this end, we conducted a comprehensive systematic review to better understand and analyze the role of spirituality in neuro-oncological settings. In this way, we highlight how spirituality may be relevant from patient, family, and care team perspectives, and may be utilized to improve personal coping, resilience, and mental well-being in the face of daunting neuro-oncologic diagnoses.

## 2. Materials and Methods

### 2.1. Literature Search and Methodology

PUBMED/MEDLINE was queried on 7 May 2024 with a search strategy to include all studies examining spirituality in a neuro-oncological setting. A broad search was conducted including search terms such as “spirituality”, “holistic medicine”, “brain tumors”, “neuro-oncology”, and “glioma” (see Table 1). Following retrieval, 214 articles were uploaded into Covidence Review Software for further screening. No duplicates were identified. Articles were then screened for topical relevance.

### 2.2. Screening and Data Extraction

**Figure 1:**
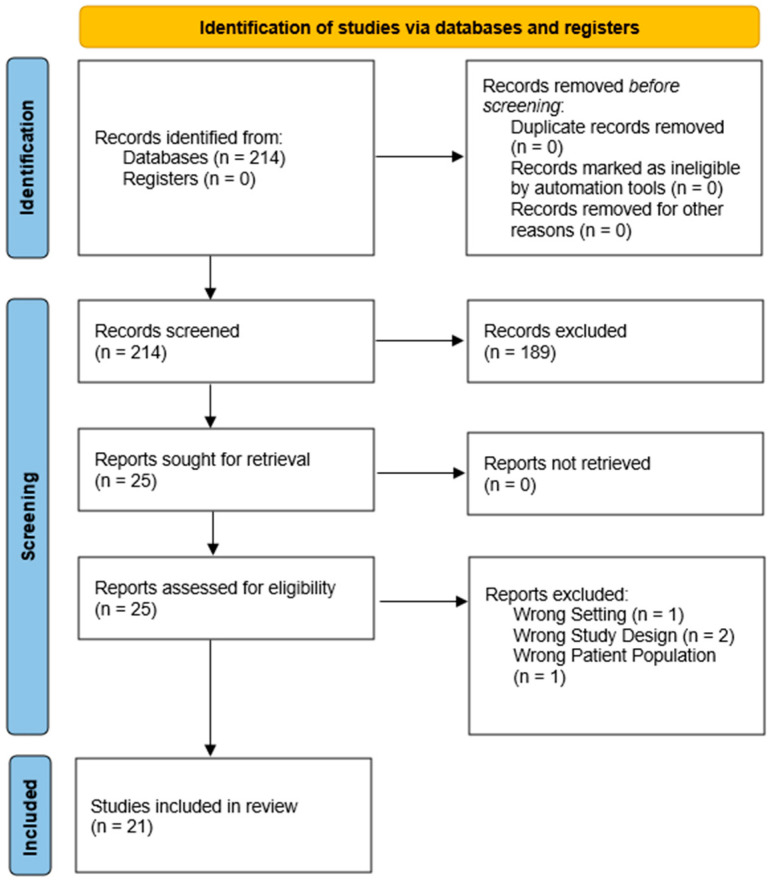
PRISMA guidelines [12] highlighting systematic inclusion and exclusion of articles at various stages.

All retrieved articles were screened by two independent review authors (NHM and KO) at the title/abstract level. Literature reviews, systematic reviews, meta-analyses, recommendations, and other article types without primary data were excluded at this stage. Manuscripts were reviewed for primary data regarding patient, family, or provider perspectives on spiritual concerns and well-being in the setting of any brain tumor. Interventional studies, case reports, surveys, and interview-based studies were included in this study. Full texts were retrieved for all remaining articles and a second round of screening was conducted by two independent review authors (NHM and KO). Inclusion criteria included randomized controlled trials, interviews, surveys, or case reports examining the relevance of spirituality and spiritual well-being, as defined by the study authors, to neuro-oncological clinical care, quality of life, or patient experience. Exclusion criteria included studies that did not directly assess perspectives on spirituality, patients with neurological diagnoses other than intracranial tumors, and papers without primary data (systemic reviews, literature reviews, conference abstracts). Inclusion and exclusion of studies was conducted in accordance with PRISMA guidelines (see Figure 1). All conflicts that arose at either level of screening were resolved via consensus of both reviewers.

### 2.3. Data Synthesis and Analysis

Included studies were examined, and details including study design, participants (patients, caregivers, healthcare providers), sample size, and key findings were extracted. Given the large heterogeneity in study design, ranging from interventional studies to interview-based analyses of patient and family experiences, and the variation in the primary diagnoses and outcomes reported, the studies were narratively synthesized.

## 3. Results

### Search Results

A comprehensive PUBMED/MEDLINE search yielded a total of 214 articles. At this stage, no articles were removed as duplicates. Screening at the title–abstract level excluded 189 studies, yielding 25 papers for retrieval and further examination. All 25 articles were assessed for eligibility at the full-text level, of which 21 studies were ultimately included in this study. Overall, the vast majority of studies were of qualitative study design and relied on one-on-one interviews or surveys. Eight studies focused only on patients, while two studies evaluated only hospital staff, and four involved only caregivers. The remaining studies (*n* = 7) included mixed study samples with patients, families, or hospital staff. Additionally, the studies included here encompassed 1985 patients, 359 caregivers, and 48 hospital providers. The included studies are characterized in Table 2 and narratively synthesized below.

There was a general consensus among patients and caregivers that brain tumors are a uniquely difficult disease within the realm of malignancies [13,15,20]. In this setting, spiritual beliefs may help patients and their families make sense of a condition that otherwise appears random, dangerous, and scary. Spiritual distress can be met through pastoral and spiritual care techniques that enhance the patient’s spiritual well-being. Numerous studies highlighted the reliance of patients or their caregivers on spirituality, a concept considered grander and more unifying than religion, which helped in their understanding and experience of disease [13,15]. This may be particularly important in understanding how spirituality, independent of organized religion and religious practices, can serve as a personal and subjective mediator of wellness. Multiple studies have reported that patients and caregivers expressed desires for spiritual care from healthcare providers but often felt that these needs were poorly met [16,23,29,32], which may be in part attributable to a lack of training [15] and time [20,23] in these care settings. Taken together, the included studies suggested that spiritual care may improve the quality of life and mental wellness of patients and caregivers, but its implementation or integration into clinical care requires further evaluation.

## 4. Discussion

Medically relevant spirituality is perhaps best understood as a personalized belief system that aims to ascribe meaning or connection to a dynamic life that has been impacted by disease and illness [34]. Indeed, there have been multiple impactful and carefully dissected interpretations of spirituality in clinical settings [35,36,37]. Consensus from multiple international groups defines spirituality with common features, with one definition characterizing spirituality as “the aspect of humanity that refers to the way individuals seek and express meaning and purpose and the way they experience their connectedness to the moment, to self, to others, to nature, and to the significant or sacred” [37]. As such, spiritual interactions focus on encouraging patients and their families to maintain hope and build resilience [17], two key elements that have been directly associated with a patient’s decision to remain engaged with their own healthcare and with improved health outcomes and quality of life [38]. Spirituality has been identified in patients with primary brain tumors as a significant predictor of “psychological hardiness” [31], defined by Kobasa in the late 1970s as a measure of the “source of resistance to stressful events in life” [39]. Despite this, there has been limited examination of spirituality and spiritual care in the management of brain tumor patients, and nearly no systematic reviews of the evidence. To that end, our findings point to the important role that spirituality has the potential to play in patients, caregivers, and provider experiences of clinical care.

### 4.1. Patient

Methods to take care of one’s spiritual well-being can fall into the realm of complimentary and integrative health interventions. One study that sought to identify all the different interventions that brain tumor patients utilized found that praying and spiritual healing were among the most popular [28]. However, they could not determine if there was a causative improvement in self-reported quality of life. They did identify that the patient should share all interventions with their healthcare team to foster open communication and allow for better development of a personalized treatment plan that will work best for the patient [28].

While praying may have been a significant strategy for patients with cancer, patients with brain cancers tend to focus more on their “self” rather than the idea that a metaphysical force (God, divine energy, etc.) is responsible for their condition and healing [13,15,17,26]. For example, one study highlighted how psychological hardiness can be increased in patients dealing with situations that induce great stress and conflict in one’s life. Psychological hardiness involves developing a personality that allows one to be resilient in difficult situations such as the diagnosis of a brain tumor. The study concluded that, as spirituality increased, patients demonstrated correlated improvements in psychological hardiness, making them better equipped to handle their diagnosis [31]. This study, however, was limited by its cross-sectional design, and further longitudinal evaluations are needed to understand how spirituality may drive psychological and emotional stability throughout the disease course.

While psychological hardiness provides self-resilience to the patient, there are other aspects of spiritual care such as patients’ relationships with loved ones that should also be addressed. For example, patient perspectives reveal that spiritual concerns are driven by a loss of independence and control over one’s life [32]. As patients are no longer able to engage in basic elements of their previous lifestyle, they struggle with relationships when they become disconnected from their spirituality and personal meaning. The source of this disconnection may be attributed to a feeling of inadequacy, since patients are no longer able to spiritually lead and guide their loved ones. Addressing this issue with pastoral care interventions via the help of a trained spiritual leader as well as the support of loved ones can affirm patient emotions and address the source of their spiritual distress. A study characterizing pastoral care utilization across a broad range of cancer diagnoses revealed that patients with a late-stage cancer diagnosis or a poor-prognosis brain cancer are more likely to use pastoral care services [29]. In these situations, spiritual distress is correlated with worse clinical outcomes, and pastoral care interventions as part of a multidisciplinary approach to treatment can serve to alleviate these stressors and equip patients with the inner resources and support system needed to address their spiritual needs. The most common of these interventions in brain cancer patients included verbalizing feelings and sharing hopes or fears [29]. In one specific case, the integrated nature of a spiritual advisor in the medical care of a patient was demonstrated via first-person qualitative analysis of perspectives from a patient and their spiritual advisor regarding the benefits of addressing spiritual needs during brain tumor management [14]. The patient stated that the spiritual care provided by the advisor allowed him to complete psychological treatment and significantly improve his quality of life by finding meaning and understanding [14]. On a broader level, one study involving 845 patients with brain tumors found that those who scored high on the Functional Assessment of Chronic Illness Therapy-Spiritual Well-Being 12 (FACIT-Sp-12) subscales of meaning, peace, and faith had a greater spiritual well-being, translating to better self-reported health-related quality of life [30]. These findings have been reaffirmed by caregivers following the death of their loved ones, who reported greater acceptance and dignity in near-death clinical states with better spiritual well-being [21].

Identifying novel interventions to improve spiritual well-being in patients and subsequently caregivers and providers is critical to improving patients’ reported quality of life. One multidimensional model assessed the perceived quality of life in 199 patients with chronic brain pathologies in correlation with spiritual markers (like meaning of self, independence, and transcendence) [27]. Here, spiritual facets of life such as inner independence, completeness, kindness, acceptance, and openness cooperatively predicted quality of life to a greater degree in patients with brain cancer than healthy controls. Further, positive spiritual experiences utilizing “inner resources” such as awe, openness, hope, and optimism were able to provide personal meaning to patients, reinforce spiritual coping in disability, and encourage adherence to clinical treatment [27]. Applied practically, a pilot study titled “Hear My Voice” at the Mayo Clinic found that recording and sharing the patient’s spiritual identity with loved ones improved the patient’s spiritual well-being in the realms of peace and existence [22], allowing the patients to access their spiritual resources and improve their physical and spiritual well-being. Further, spiritual care provided by a trained chaplain also benefitted caregivers and hospital staff, providing them with the necessary tools and information to comfortably engage in spiritual discussions and support with patients [22,25]. Providing similar interventions at follow-up visits for patients and their caregivers also demonstrated an increase in the peace subscale of the FACIT-Sp-Ex, indicating that carefully executed spiritual care interventions can improve peacefulness and positive spiritual coping for patients and their caregivers [24].

More recently, “dignity therapy” (DT), involving reflection on a patient’s life, analyzing sources of pride, and developing a “legacy document” for each patient, has been utilized effectively in patients with brain tumors [40]. The development and implementation of dignity therapy further underscores the potential of holistic care interventions for patients with brain tumors.

### 4.2. Family or Care Givers

Overall, caregivers tend to place a greater importance on spirituality in reasoning and coping with the effects of illness than patients [13,15,17,26]. Of the coping strategies used, spiritual coping was among the most prevalent [26], with many caregivers identifying spiritual strength through hope, meaning, or understanding as critical to helping them persevere through their loved one’s brain tumor [15,17]. In many cases, interviews conducted to gauge caregiver experiences noted that the interview, in and of itself, was therapeutic, largely given the recognition of a shared experience and the access to a space to share and express themselves [14,15,17,32]. Further, the reduction in time devoted to regular spiritual activity to care for their loved one is an often overlooked contributor to burnout, which can exacerbate spiritual distress for the caregiver [32]. Tailored spiritual interventions may help address these concerns, as exemplified by one study that implemented mean-centered psychotherapy (MCP-C) for caregivers, and found significant improvements in personal meaning and existential well-being [33]. MCP-C also improved the overall spiritual well-being and “sense of faith” in caregivers, which lasted nearly 2 months post intervention [33], a critical period of time when caring for a terminally ill patient. Similarly, another study reported fewer depressive symptoms 8 months into caring for a patient in caregivers with a higher level of spirituality than caregivers with lower levels of spirituality [19]. This suggested that provider screening of caregivers prior to and during the care of the patient in regard to distress and coping resources may be useful to identify maladaptive coping methods [26]. Altogether, caregiver burnout and existential distress are indeed concerns in neuro-oncological care and warrant special attention and targeted interventions.

### 4.3. Provider

Overall, there is some evidence of the provider’s role in delivering or engaging with spiritual care for the neuro-oncologic patient. Firstly, there is an immense potential to deepen the therapeutic alliance between an individual provider and their patient suffering from brain cancer when addressing the spiritual beliefs of the patient [15]. This therapeutic alliance was exemplified by nurses caring for brain tumor patients who talked and listened to patients’ spiritual concerns, building a relationship with the patient that extended past social niceties [20]. There is agreement in the literature that some level of training in spiritual care must be given to adequately meet the patient’s spiritual care needs [15,20,29,31]. The utility of providing spiritual care in the acute neuro-oncological setting, however, may not be as effective as in the palliative care setting given the reduced length of time in caring for patients with dangerous CNS neoplasms [20,23]. Additionally, while providers may not have the time to address spiritual components of care, many may also be hesitant to address these concerns given emotional burnout or lack of experience and comfort [23]. Nonetheless, patients and caregivers have expressed desires for their healthcare providers to engage in discussions related to spiritual care including meaning-making, existential concerns, and end-of-life discussions. As such, physicians should attempt to integrate modalities used by patients to improve their spiritual well-being into treatment plans [28]. Notably, a lack of psychosocial support from clinicians during the time of treatment was distressing for patients, suggesting that involvement from providers is crucial when addressing the multidimensional concerns of patients with dynamic existential trajectories [18]. The gradual progression of disease towards physical decline and the unconfirmed timeline of fatality created questions of meaning and value for patients and caregivers, which necessitated support and reassurance from healthcare providers. Many providers were willing to address these psychosocial needs as a part of their role and responsibility, while others felt they should be limited to their clinical duties of providing medical information and controlling symptoms [18]. The desire to engage healthcare professionals who are limited by clinical responsibilities and time may be accomplished through increasing the importance of a spiritual advisor in the regular treatment plan of the patient fighting the brain tumor or finding small fixes such as “presencing” or fostering more patient family visits by the healthcare staff [16]. Further work to better understand how physician training, education, and clinical practice can adapt to incorporate spiritual care and awareness when needed may help improve patient quality of life and deepen the patient–physician relationship, particularly in settings of clinical distress such as brain tumors.

### 4.4. Future Directions and Limitations

Despite compelling evidence highlighting the relevance of spiritual awareness in neuro-oncologic care, there remain several significant gaps in its integration within clinical practice [28]. Inevitably, some of these gaps likely reflect the lack of universal definitions related to patients’ spiritual needs, or a lack of appreciation for the role that surgeons and other non-pastoral providers can play in bridging these gaps [6]. Indeed, there is a particular dearth of studies examining how spiritual awareness among neurosurgeons or neuro-oncologists can interact with clinical care, and there have been disproportionately fewer studies in the neuro-oncological patient population relative to alternative specialties such as interventional cardiology. Additionally, many of the studies included in this systematic review were retrospective cohort studies that harbor natural design limitations in their evaluations of spirituality across clinical settings. More comprehensive assessments of spiritual care interventions in neuro-oncological settings require prospective and longitudinal evaluations. However, it is unclear at this stage how spiritual care can be best addressed in patients with brain tumors and their families, and further longitudinal and educational work may be needed to optimize clinical outcomes while fostering a complete sense of healing.

The integration of spirituality into neuro-oncological education across levels of training may represent a key untouched frontier that can help optimize clinical care, deepen comfort with difficult situations, and stimulate growth of the patient–physician relationship. In one example, nurses working with neuro-oncological patients generally identified the spiritual needs of patients and families, including “showing sensitivity to patients’ emotions”, “responding to religious needs”, and “supporting them with end-of-life care”, but often did not feel adequately prepared to effectively respond to these needs [20]. Similarly, from the patient’s perspective, there were some reports expressing need for spiritual help that was not adequately addressed by members of the care team [20]. Further exacerbated by the relative lack of emphasis within medical education and the high burden of clinical responsibility placed on physicians and physician trainees, this spiritual disconnect may be consolidated as a function of the often hectic, intense, and stressful environment of the in-patient and surgical spheres [14]. Indeed, one cohort of children with brain tumors and their families reported nearly unanimous satisfaction with their medical care, but demonstrated the urge for more “existential support” [23]. While spirituality may be a relatively minor component of the hospitalized surgical patient’s lived experience and quality of life, equipping providers with the knowledge, skills, and sensitivity necessary to help patients and their families correctly set perspectives, maintain realistic hope, and build resilience is a fundamental skill that requires further evaluation. To that end, further recognition of the spiritual, existential, and cultural dimensions of neuro-oncological care and an expanded evaluation of how these components can be integrated into medical, residency, and fellowship training are warranted.

Importantly, there are several limitations to this study that deserve mention that largely anchor on methodological constraints, gaps in the literature, and the difficulty of examining spirituality in neuro-oncological settings. Firstly, all studies identified here display significant heterogeneity in design, interventions, outcome reporting, and, perhaps most importantly, definitions of spirituality and spiritual awareness. To that end, a standardized definition of spirituality becomes quite difficult, and its effect on and relevance to patients, providers, and family may be confounded by diverse and personalized interpretations. Similarly, this study did not comprehensively identify, assess, or meta-analyze the relationship between spirituality and functional status or cognitive state. A better understanding of these relationships can be developed via targeted assessments of spiritual care interventions across diverse patient populations with heterogenous functional capacity. Further, this study included only articles published in the English language, limiting the comprehensive appraisal of spirituality in a global context. Given regional, cultural, and religious variations in definitions and interpretations of spirituality and holistic medicine, further work is needed to probe the global landscape of spiritual awareness as a medical tool to unite providers with patients and their families. Additionally, there exist very few primary interventional randomized trials that attempt to implement spiritual or pastoral care interventions of any kind in neuro-oncological settings, drastically limiting their broader application. Further longitudinal and randomized interventional trials are needed to expand our understanding of spiritual awareness in neuro-oncological care beyond prevalence surveys and quality-of-life analyses. Finally, the scope of this study was limited to the examination of spirituality for the neuro-oncologic patient and caregiver, and as such does not address its efficacy or relevance to other acute or chronic neurological diseases. Further longitudinal work involving diverse patient populations and culturally sensitive and inclusive definitions of spirituality may further advance our understanding of spirituality’s influence on patient and caregiver mental wellness, optimizing its integration into clinical diagnosis and longitudinal management.

## 5. Conclusions

Spirituality may be an important component of holistic healthcare in patients with brain tumors, as well as their families, that has the potential to improve quality of life and mental wellness and deepen the patient–physician relationship. As such, spiritual care interventions warrant further attention and education. At this time, however, it is unclear how spiritual care and awareness can best be integrated into clinical education and practice. Further work to better appreciate the components of spiritual care and their relevance to neuro-oncology can function to optimize the patient experience, improve mental well-being and quality of life, and further improve the patient–physician relationship.

## Figures and Tables

**Table 1:** PUBMED search strategy.

Database	PUBMED/MEDLINE
**Search String**	(Spirituality OR Holistic Medicine) AND (Brain Tumors OR Neuro-oncology OR Glioma OR meningioma OR astrocytoma OR glioblastoma OR ependymoma OR schwannoma OR pituitary adenoma OR oligodendroglioma)
**Date Queried**	7th May 2024
**Articles Retrieved**	214

**Table 2:** Summary of 21 included articles highlighting the relevance of spirituality to patients, families, and hospital staff.

Study	PMID	Design	Participants	*n*	Key Findings
Strang et al. 2001 [13]	11301663	Qualitative Study (Interviews)	Patients	20	Most patients and their loved ones did not follow traditional religion; however, they reported a spiritual experience of their disease consisting of elements of faith including life after death, destiny, and the goodness of life. Coping strategies involve elements of spirituality including hope, redefinition of illness, rationalization, meaningfulness, and close relations with loved ones.
			Caregivers	16	
Brody et al. 2004 [14]	15226285	Case Report	Patient	1	A narrative writing intervention implemented in a patient with spinal cord injury and astrocytoma improved their ability to attribute meaning to their medical story. Spiritual counseling and holistic care provided by a family physician, spiritual counselor, and psychologist helped address core psychological concerns by developing inner strength to manage their illness.
Lipsman et al. 2007 [15]	17996072	Qualitative Study (Interviews)	Patients	7	For patients and their loved ones, discussing their conditions enabled the discovery of inner strength and resilience to better navigate their difficult disease trajectories. Most patients and their caregivers reported improved navigation of their illness after engaged discussions revolving around spirituality, quality of life, and mental competence.
			Caregivers	22	
Nixon et al. 2010 [16]	20529167	Qualitative Study (Survey)	Patients	21	Patients diagnosed with brain tumors reported expressing spiritual needs in the hospital setting that were not always adequately acknowledged. As identified by patients, successful strategies included the use of healthcare professionals to provide existential support, flexibility with hospital policies, encouragement with family relationships, and, at times, religious support.
Zelcer et al. 2010 [17]	20194254	Qualitative Study (Interviews)	Caregivers	25	Parents of children dying of brain tumors identified “maintaining normalcy” and spiritual strength for hope as key coping mechanisms in managing their child’s disease. Struggles for these parents involved balancing responsibilities, financial/practical hardships, and discussing the implications of death with their children.
Cavers et al. 2012 [18]	22431898	Prospective Qualitative Study (Interviews)	Patients	26	Patients and their families reported experiencing social and emotional duress prior to diagnosis, with psychological and existential distress during diagnosis and treatment. Improved understandings of psychosocial and spiritual components of patient well-being may help clinicians predict patients’ disease course, allowing for conscious and personalized communication.
			Caregivers	23	
			Hospital Staff	19	
Newberry et al. 2013 [19]	23615145	Prospective Qualitative Study (Interviews)	Patients	50	For both primary malignant tumor patients and their loved ones, spirituality remained a stable trait across disease courses. Spirituality was statistically correlated with lower depressive (*p* < 0.01) and anxiety (*p* < 0.01) symptoms for patients and their families and also served as a protective barrier against poor mental health outcomes.
			Caregivers	50	
Nixon et al. 2013 [20]	23374999	Mixed Methods (Surveys + Thematic Analysis)	Hospital Staff	12	Neurosurgical nurses reported that they are aware of their patients’ spiritual needs, while also encouraging discussions about their feelings and helping them explore sensations of despair, meaninglessness, and end-of-life care. Some nurses expressed not always feeling ready or equipped to meet the spiritual needs of their patients.
Sizoo et al. 2014 [21]	24162875	Retrospective Qualitative Study (Survey)	Caregivers	83	Quality-of-life evaluations from caregivers of deceased patients revealed a significant burden of physical and psychosocial deterioration during disease course. Existential realizations including dying with dignity and recognition of death tended to increase as the patient approached end of life, suggesting that the end-of-life period may be particularly important to existential QoL.
Piderman et al. 2015 [22]	24952300	Prospective Qualitative Study (Interviews)	Patients	25	Hear My Voice, a spiritual intervention for patients with brain tumors, gave them the ability to use their voice to document their spirituality, allowing them to redefine their journey and provide meaning to their condition. The use of Hear My Voice for patients allowed for a patient-centered plan of care, with the potential to dramatically improve clinical practice.
Strang et al. 2001 [23]	11762974	Qualitative Study (Interviews)	Patients	20	Neuro-oncological patients and their families identified receiving existential support from hospital staff as important to their personal comfort and ability to speak about their diagnoses. Hospital staff recognized the importance of addressing existential concerns in patient care, but were constrained by time, knowledge, and fear.
			Caregivers	16	
			Hospital Staff	16	
Piderman et al. 2017 [24]	26643586	RCT	Patients	24	Patients and their families participating in a chaplain-directed spiritual interview intervention demonstrated improved levels of spiritual well-being, quality of life, and positive coping mechanisms.
			Caregivers	24	
Piderman et al. 2017 [25]	27398684	Prospective Qualitative Study (Interviews)	Patients	19	Chaplain-led spiritual interviews in patients with brain cancer was reported to improve their spiritual understanding, with beneficial effects on their relationship with stress and grief.
Cutillo et al. 2018 [26]	30485195	Prospective Qualitative Study (Interviews)	Caregivers	40	Caregivers of children who recently underwent brain tumor surgery reported relying on spiritual, emotion-focused, and social support coping mechanisms, but were at increased risk of “maladaptive” coping techniques. Improved identification of coping mechanisms in these parents can help employ healthy adaptative coping strategies and reduce psychological burden.
Giovagnoli et al. 2019 [27]	30851485	Comparative Cohort Study	Patients	28	A factor-based survey analysis revealed that spirituality, characterized by meaning-making, inner independence, and transcendence, and emotional factors were cooperatively associated with quality of life in patients with brain tumors or neurodegenerative diseases. A multidimensional disease model that involves spiritual and emotional components may better characterize quality of life in these patients.
Randazzo et al. 2019 [28]	31383442	Retrospective Cohort Study	Patients	845	Nearly 76% of patients with brain tumors reported relying on integrative health modalities, which frequently included spiritual healing, prayer, diet, meditation, vitamins, and massage.
Hyer et al. 2021 [29]	32799646	Retrospective Cohort Study	Patients	232	Pastoral care services were widely utilized by cancer patients, particularly for brain, liver/pancreas, and lung cancer. Patients with advanced stages of cancer were identified as having greater likelihoods of receiving pastoral care services, highlighting its importance as a treatment approach for cancer.
Randazzo et al. 2021 [30]	34055377	Retrospective Cohort Study	Patients	606	Overall, 45% of brain tumor patients reported prayer as a component of their faith, and reported relying on it for hope or as a coping skill. Greater spiritual well-being was associated with an elevated health-related quality of life in these patients.
Baksi et al. 2021 [31]	33818705	Prospective Cohort Comparisons	Patients	61	Patients with brain tumors were found to have statistically lower mean scores of spirituality and psychological hardiness (*p* < 0.001). Improved spirituality scores and age were significant predictors of psychological hardiness, suggesting that the poor psychological toll of brain tumors may be targeted with spiritual care.
			Healthy Subjects	61	
Sprik et al. 2021 [32]	32921085	Qualitative Study (Interview)	Hospital Staff	1	Patients with brain cancer identified religion and spirituality as key components of their coping mechanisms in managing their diagnoses. Hear My Voice, a spiritual care intervention, improved patients’ ability to talk about their beliefs, practices, values, struggles, and life-learned wisdom in the setting of their diagnoses.
Appelbaum et al. 2022 [33]	35852487	Mixed-Methods RCT	Caregivers	60	Meaning-Centered Psychotherapy (MCP-C) for cancer caregivers addressed existential distress in caregivers of patients with glioblastoma multiforme Outcomes of parents receiving MCP-C included improvements in spiritual well-being, depressive symptoms, and meaning-making.

## Data Availability

All papers identified in this manuscript are peer-reviewed and publicly available.
